# Genes and hormones of the hypothalamic–pituitary–adrenal axis in post-traumatic stress disorder. What is their role in symptom expression and treatment response?

**DOI:** 10.1007/s00702-021-02330-2

**Published:** 2021-04-07

**Authors:** Susanne Fischer, Tabea Schumacher, Christine Knaevelsrud, Ulrike Ehlert, Sarah Schumacher

**Affiliations:** 1 Institute of Psychology, Clinical Psychology and Psychotherapy, University of Zurich, Binzmuehlestrasse 14/Box 26, 8050 Zurich, Switzerland; 2 Department of Education and Psychology, Division of Clinical Psychological Intervention, Freie Universität Berlin, Berlin, Germany; 3 Health Faculty, Clinical Psychology and Psychotherapy, Health and Medical University Potsdam, Potsdam, Germany

**Keywords:** Cognitive behavioural therapy, Cortisol, Methylation, Post-traumatic stress disorder, Psychotherapy

## Abstract

**Background:**

Less than half of all individuals with post-traumatic stress disorder (PTSD) remit spontaneously and a large proportion of those seeking treatment do not respond sufficiently. This suggests that there may be subgroups of individuals who are in need of augmentative or alternative treatments. One of the most frequent pathophysiological findings in PTSD is alterations in the hypothalamic–pituitary–adrenal (HPA) axis, including enhanced negative feedback sensitivity and attenuated peripheral cortisol. Given the role of the HPA axis in cognition, this pattern may contribute to PTSD symptoms and interfere with key processes of standard first-line treatments, such as trauma-focused cognitive behavioural therapy (TF-CBT).

**Methods:**

This review provides a comprehensive summary of the current state of research regarding the role of HPA axis functioning in PTSD symptoms and treatment.

**Results:**

Overall, there is preliminary evidence that hypocortisolaemia contributes to symptom manifestation in PTSD; that it predicts non-responses to TF-CBT; and that it is subject to change in parallel with positive treatment trajectories. Moreover, there is evidence that genetic and epigenetic alterations within the genes *NR3C1* and *FKBP5* are associated with this hypocortisolaemic pattern and that some of these alterations change as symptoms improve over the course of treatment.

**Conclusions:**

Future research priorities include investigations into the role of the HPA axis in day-to-day symptom variation, the time scale in which biological changes in response to treatment occur, and the effects of sex. Furthermore, before conceiving augmentative or alternative treatments that target the described mechanisms, multilevel studies are warranted.

## Introduction

Post-traumatic stress disorder (PTSD) is a pathological response to a traumatic event involving exposure to actual or threatened death, serious injury, or sexual violation (APA 2013). The prevalence rate of PTSD lies at 2.3%, with a clear female preponderance (3.6% vs. 0.9% in men; Jacobi et al. 2014). The twelve-month incidence rate is particularly high after interpersonal trauma, with 37.5% of individuals developing PTSD after being raped and 35.5% following sexual abuse during childhood, compared to 6.9% after witnessing traffic accidents or violence (Maercker et al. 2008). Individuals affected by PTSD experience a wide range of distressing symptoms, such as flashbacks, negative affect and thoughts, feelings of isolation, and difficulties concentrating or sleeping (APA 2013). The experience of such symptoms is known to impair one’s ability to participate in social activities or relationships (Geisser et al. 1996) or to work regularly (Matthews 2005; Smith et al. 2005), and the risk of suicidal behaviours is increased two- to three-fold in this population (Kanwar et al. 2013; Bentley et al. 2016). Moreover, PTSD exerts a significant economic burden on society. In Europe alone, the annual healthcare costs are estimated at 8.4 billion Euros, with 7.7 million individuals currently affected (Gustavsson et al. 2011).

As shown in a meta-analysis, on average, only 44% of individuals with PTSD remit spontaneously after more than 3 years (Morina et al. 2014). Fortunately, a number of treatments are readily available to prevent chronic illness trajectories. Among these, trauma-focused cognitive behavioural therapy (TF-CBT) is the gold standard for PTSD (Lee et al. 2016). It comprises behavioural interventions, such as prolonged exposure to traumatic cues or memories (Foa 2007), as well as cognitive interventions, such as the cognitive restructuring of dysfunctional trauma-related beliefs (Resick et al. 2007). Given that PTSD often affects individuals with limited access to such treatments (e.g., less mobile elderly people, rural populations), there has been a growing interest in internet-based administrations of such treatments. These are usually therapist-guided, and have been shown to be effective in numerous different populations, such as patients who have suffered pregnancy loss, intimate partner violence, or rape (see Sijbrandij et al. 2016; Kuester et al. 2016 for meta-analyses). However, despite the general effectiveness of both face-to-face and internet-based TF-CBT, some studies have reported substantial non-responder rates (Ponniah and Hollon 2009). This implies that a large proportion of patients with PTSD remain symptomatic, with an increased risk of a chronic progression of the disorder.

A crucial first step towards the prevention of a protracted course of the disorder is to identify factors that contribute to PTSD perpetuation and non-responses to standard first-line treatments. The most frequently studied candidate factors to date are PTSD symptom severity, trauma type, and comorbidity with other mental disorders (Schottenbauer et al. 2008; Morina et al. 2014). The problem with these clinical characteristics is that they are limited in their ability to shed light on why PTSD persists in some individuals but not in others. However, evidence illuminating the mechanisms underlying this phenomenon is now beginning to accumulate.

## Hypothalamic–pituitary–adrenal axis functioning

Post-traumatic stress disorder has been found to present with alterations across various neurobiological systems (Yehuda 2002). Given its aetiology, a major research focus has been on the stress-responsive hypothalamic–pituitary–adrenal (HPA) axis and its end product, the glucocorticoid cortisol (Ehlert et al. 2001). Cortisol can be measured non-invasively by repeated saliva sampling at fixed time points over the course of at least two consecutive days (Stalder et al. 2016). Five meta-analyses have provided evidence that a substantial subgroup of individuals with PTSD are characterised by hypocortisolism, that is, reduced levels of cortisol (Morris et al. 2012; Meewisse et al. 2007; Schumacher et al. 2019; Pan et al. 2018, 2020). A common explanation for this phenomenon is an enhanced negative feedback sensitivity (Daskalakis et al. 2013). This means that the glucocorticoid receptors (GR), which are expressed at the hypothalamic and pituitary level, are more responsive to the effects of cortisol reaching the brain, thus leading to a stronger-than-usual inhibition of the HPA axis. An important co-chaperone of glucocorticoid sensitivity is the FK506 binding protein 5 (FKBP5), which reduces the GR’s affinity to cortisol.

Importantly, this hypocortisolaemic pattern does not appear to be a mere consequence of PTSD but most likely represents a key factor underlying its development and maintenance. This is due to the key role of cortisol in fear conditioning. Numerous studies have demonstrated that cortisol facilitates the consolidation of fear memories, while at the same time impeding their retrieval (de Quervain et al. 2017). Acute increases in cortisol during traumatic situations thus support the transfer of short-term fear memories into long-term fear memories. This effect depends on the concomitant activation of the central noradrenergic system and occurs via non-genomic and genomic effects within the amygdala, hippocampus, and cortex. Following this initial consolidation, it is assumed that in some individuals, the initially elevated cortisol concentrations decrease overtime via hypersensitisation of GR (Steudte-Schmiedgen et al. 2016), resulting in the hypocortisolaemic pattern as described above. In the same individuals, a failure of cortisol to inhibit the retrieval of fear memories may then contribute to chronic re-experiencing of traumatic situations (e.g., via flashbacks). Indeed, the administration of hydrocortisone, a synthetic analogue of cortisol, has been found to reduce PTSD symptoms and incidence when administered in a preventive context (Kothgassner et al. 2021). Furthermore, in individuals with PTSD, negative associations of cortisol with symptoms of intrusion, as well as with symptoms of avoidance, numbing, and hyper-arousal, have repeatedly been reported (Castro-Vale et al. 2016). Given that these symptoms fluctuate both within and across days (e.g., Biggs et al. 2019; Schuler et al. 2019), an important question that remains to be answered is whether low cortisol levels also contribute to PTSD symptom exacerbation.

As the extinction of fear memories is a main goal of TF-CBT, a further question is whether cortisol levels modulate treatment response. There is evidence to suggest that this is indeed the case. In patients with anxiety disorders, lower cortisol during exposure therapy sessions appears to be linked to poorer responses to CBT (Fischer and Cleare 2017). As outlined in the previous paragraph, this may be explained by the fact that failure to mount and maintain sufficiently high cortisol levels during exposure to phobic stimuli prevents the consolidation of an extinction memory, which is integral to the treatment of any fear-related pathology. In PTSD, only a small number of studies have investigated pre-treatment cortisol in terms of predicting outcomes after TF-CBT (Schumacher et al. 2018). A higher cortisol awakening response (CAR) (Rapcencu et al. 2017) and a more suppressed CAR after dexamethasone intake (Nijdam et al. 2015) were found to predict greater symptom improvements. Furthermore, some studies revealed changes in cortisol as a result of treatment response. However, the direction of these changes remained ambiguous, with increases (Olff et al. 2007) as well as decreases (Gerardi et al. 2010) being observed.

**Fig. 1:**
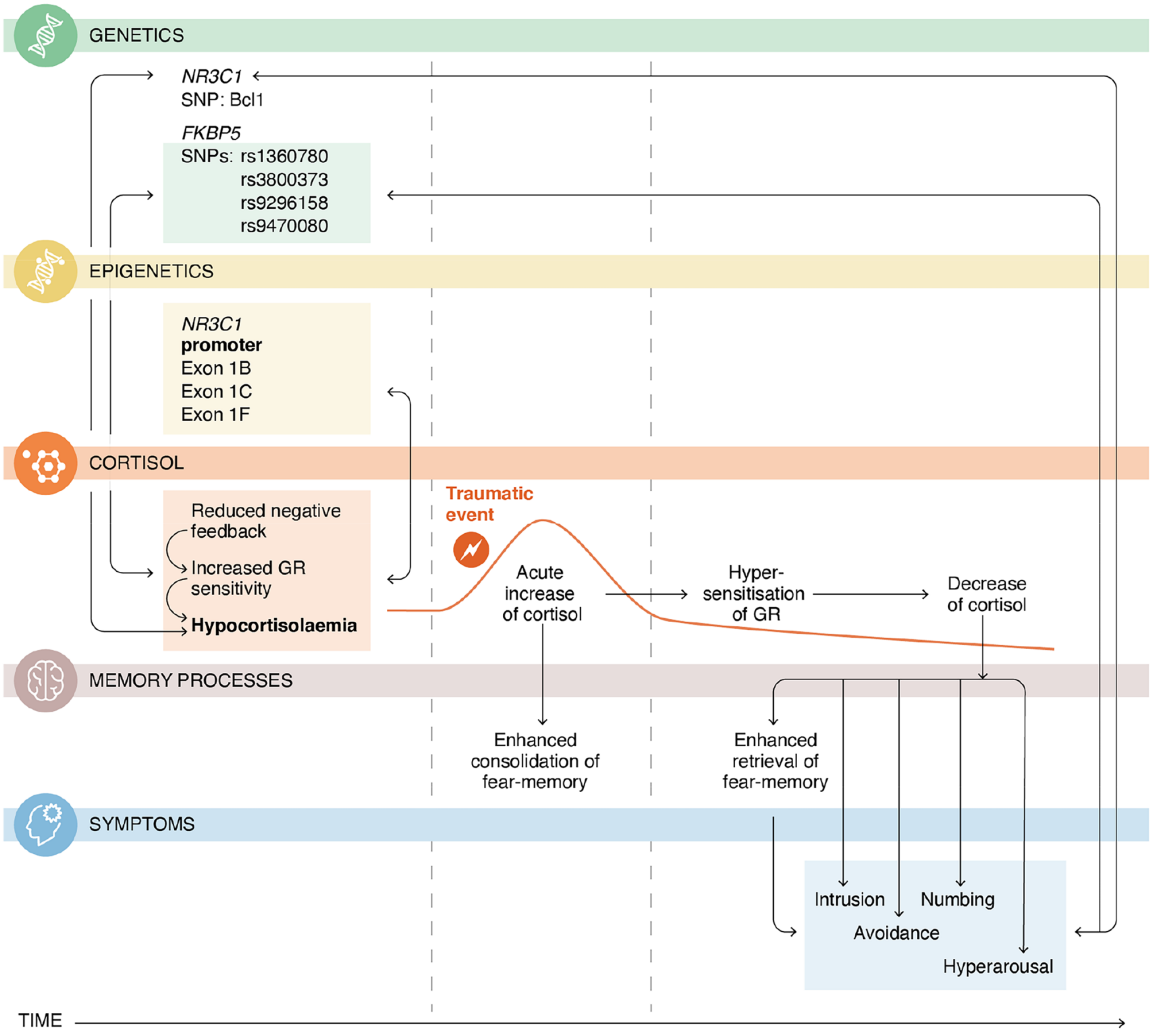
Illustration of hypothalamic–pituitary–adrenal (HPA) axis related (epi-)genetic and endocrine findings in posttraumatic stress disorder (PTSD). The current state of research suggests that at least a subgroup of individuals with PTSD presents with a number of single nucleotide polymorphisms and DNA methylation patterns that are associated with altered HPA axis functioning, such as increased glucocorticoid receptor (GR) sensitivity and hypocortisolaemia. These characteristics may directly contribute to the manifestation of PTSD symptoms via disturbed memory processes. University of Zurich, Information Technology, MELS/SIVIC, Tara von Grebel

**Fig. 2:**
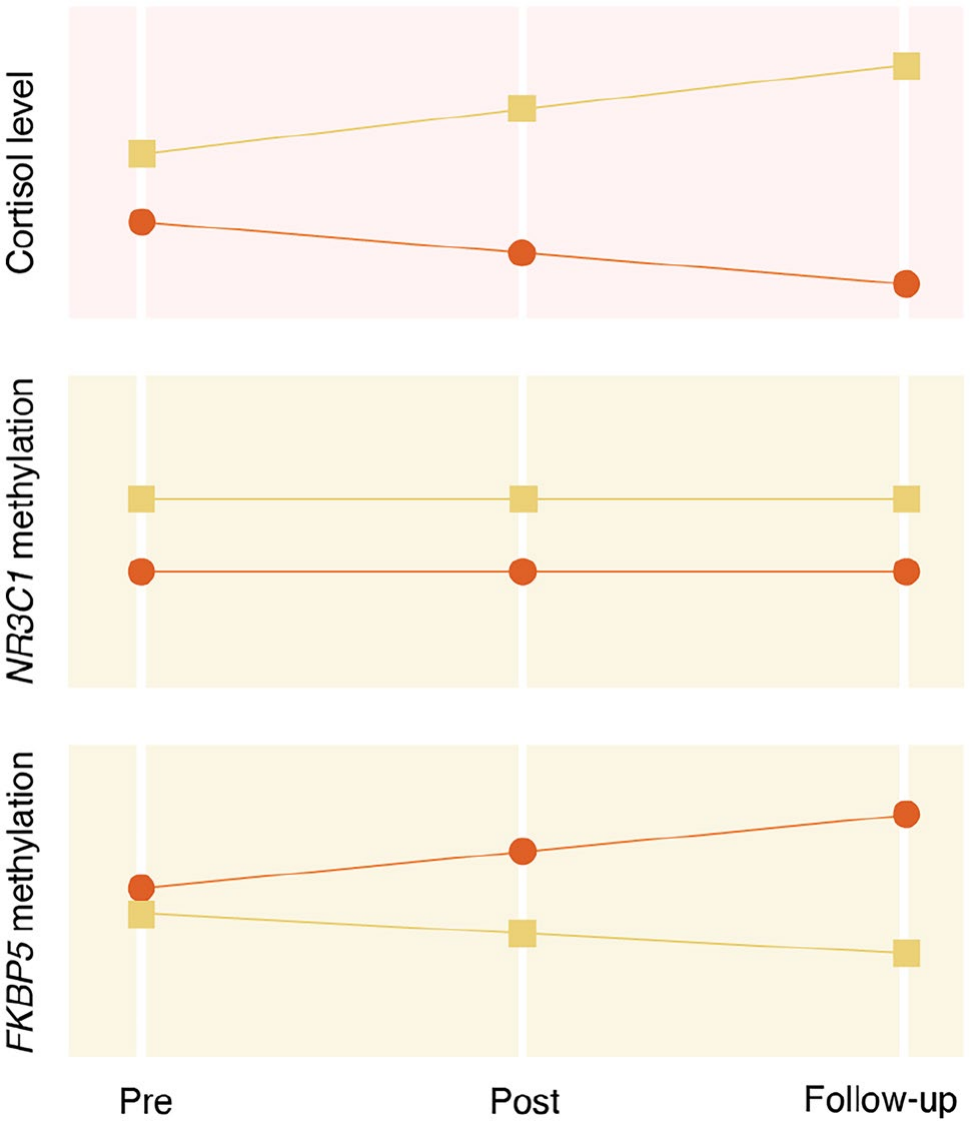
Illustration of the hypothesised relationship between hypothalamic–pituitary–adrenal axis related (epi-)genetic and endocrine markers and trauma-focused cognitive behavioural therapy (TF-CBT) for posttraumatic stress disorder (PTSD). “Pre” refers to the pre-treatment assessment, “post” to the post-treatment assessment, and “follow-up” to the follow-up assessment. Non-responders are indicated in red and responders are indicated in yellow. According to the current state of research, low levels of cortisol and high levels of *NR3C1* methylation are likely to predict non-responses to TF-CBT. Furthermore, cortisol levels presumably increase and *FKBP5* methylation decreases in parallel with positive treatment trajectories. University of Zurich, Information Technology, MELS/SIVIC, Tara von Grebel

In sum, the current state of research indicates that at least a subgroup of individuals with PTSD presents with increased GR sensitivity, which is reflected by a hypocortisolaemic pattern. Preliminary evidence suggests that this pattern contributes to the manifestation of PTSD symptoms (see Fig. 1). Moreover, the same pattern may predict non-responses to TF-CBT due to its interference with the formation of an extinction memory—and may be subject to change in parallel with positive treatment trajectories (see Fig. 2, first panel). This raises the question as to where this pattern originates in the first place.

## Genetic underpinnings—single nucleotide polymorphisms

The heritability of PTSD is estimated at between 30 and 70% (Daskalakis et al. 2018), and a number of genes known to encode different components of the HPA axis appear to be partly responsible for this. According to a systematic review of the literature, the Bcl1 single nucleotide polymorphism (SNP) within *NR3C1* and four SNPs within *FKBP5* (rs1360780, rs3800373, rs9296158, rs9470080) are particularly prominent HPA axis related risk factors for the development of PTSD (Carvalho et al. 2017). A recent meta-analysis confirmed the role of *NR3C1* and *FKBP5* in PTSD, with rs258747 and rs9296158 emerging as significant risk-enhancing SNPs (Sheerin et al. 2020). The fact that *NR3C1* is the gene encoding the GR while *FKBP5* is the gene encoding the protein of the same name resonates well with the above-outlined pathophysiological mechanism in PTSD (i.e., increased GR sensitivity). In line with this, one study found that the Bcl1 SNP within *NR3C1* was linked not only to post-traumatic symptoms but also to lower cortisol levels (Hauer et al. 2011). Similarly, rs1360780, rs3800373, rs9296158, and rs9470080 within *FKBP5* predicted both a diagnosis of PTSD and enhanced negative feedback sensitivity (Binder et al. 2008). The latter finding was complemented by further studies revealing an association of the same SNPs with cortisol (Sarapas et al. 2011; Young et al. 2018), which was mediated by FKBP5 expression (Sarapas et al. 2011). Together, these findings support the notion that individuals with a particular genetic background are more vulnerable to develop (and maintain) PTSD when faced with trauma over the course of their lifespan, and that this may occur via a functional impact of specific SNPs linked to the HPA axis.

In recent years, two studies have complemented this research by testing whether the same SNPs were predictors of treatment outcomes. In a sample of *N* = 52 war veterans with PTSD, Yehuda et al. (2014) found that those patients who were homozygous carriers of the Bcl1 C allele (*NR3C1*) were more likely to be non-responders to twelve weeks of prolonged exposure or minimal attention intervention. Focusing instead on *FKBP5*, Wilker et al. (2014) investigated *N* = 43 survivors of the rebel war in Northern Uganda and found that carriers of the rs1360780 T allele had worse long-term outcomes after 6 weeks of narrative exposure therapy. Notably, in the former study the treatment arms were collapsed for statistical analysis and the latter study only reported results for the follow-up assessments. Interestingly, the vast majority of studies published to date showed that the four SNPs within *FKBP5* mentioned above interact with childhood trauma and other environmental stressors in enhancing PTSD risk (see Wang et al. 2018 for meta-analyses; Hawn et al. 2019). The question thus arises whether environmentally induced modifications to the genome co-regulating gene expression could contribute to PTSD symptoms and treatment response.

## Epigenetic underpinnings—DNA methylation

Epigenetic research into PTSD has recently gained momentum (Zannas et al. 2015). Although not representing changes to the genetic code, epigenetic signatures, such as levels of DNA methylation or histone acetylation, are relatively stable and can be inherited. However, modifications to the epigenome can occur due to environmental influences. DNA methylation in particular has attracted researchers' attention since reports of demonstrable changes in the aftermath of childhood trauma (e.g., McGowan et al. 2009), and because it can be measured reliably in a number of tissues. In brief, DNA methylation refers to the process of methyl molecules attaching to the 5′ carbon position of cytosine residues within CpG dinucleotides. Within *NR3C1* and *FKBP5*, methylation levels are particularly variable in promoter regions, which regulate gene expression and where a number of glucocorticoid-responsive elements are located (Zannas et al. 2015). In PTSD, alterations in DNA methylation have so far been documented within the exon 1B, 1C, and 1F *NR3C1* promoter regions (Labonte et al. 2014; Vukojevic et al. 2014; Yehuda et al. 2015; Schechter et al. 2015). Again, patients not only exhibited lower methylation levels when compared to controls, but these were directly related to enhanced negative feedback sensitivity and lower cortisol concentrations (Yehuda et al. 2015; Labonte et al. 2014). It therefore seems that besides the genetic make-up, epigenetic signatures are equally relevant in HPA axis functioning in PTSD.

Only two studies to date have tested whether methylation within HPA axis-relevant genes is related to treatment responses in PTSD. In a sample of *N* = 16 war veterans, Yehuda et al. (2013) found that lower pre-treatment methylation of the *NR3C1* exon 1F promoter region was linked to poorer responses to TF-CBT. Additionally, positive outcomes were paralleled by decreases in methylation of the *FKBP5* exon 1 promoter region over the course of treatment. In a more recent study, Bishop et al. (2018) reported that changes in methylation of the *FKBP5* intron 7 region interacted with responder status after mindfulness-based stress reduction for veterans with PTSD. More specifically, the authors observed significant decreases in methylation in treatment responders (*n* = 11) and increases in methylation in non-responders (*n* = 11). The latter results suggest that methylation of *FKBP5* is malleable in that short-term changes due to environmental influences (i.e., TF-CBT) may occur. In line with this, it was found that methylation levels of intron 7 within *FKBP5* decreased over the course of only one week in N = 34 healthy university students undergoing stress management training (Stoffel et al. 2017, 2020).

Taken together, methylation of *NR3C1* may thus be considered as a prognostic marker of TF-CBT response, while methylation of *FKBP5* may be considered as a marker of symptom change (see also Fig. 2, second and third panel).

## Conclusions

In sum, the current state of research suggests that both genetic and epigenetic markers within *NR3C1* and *FKBP5* are associated with the observed hypocortisolaemic pattern as frequently present in PTSD and preliminary evidence suggests that the same markers are indicative of treatment outcomes. However, a number of gaps are still apparent in this literature and should be addressed by future research.

First, despite evidence from laboratory studies that cortisol levels are associated with PTSD symptom severity, it currently remains unknown whether diurnal changes in cortisol levels map onto PTSD symptom exacerbation in daily life. In other research domains such as depression, initial studies have, for instance, demonstrated a positive correlation between daily cortisol levels and rumination (Huffziger et al. 2013). Knowing more about the extent to which cortisol contributes to symptom exacerbation may not only provide further evidence for a role of cortisol in the pathophysiology of PTSD, but may also help patients to manage their symptoms by deliberately engaging in activities that are capable of adjusting cortisol, such as stress management strategies (Hammerfald et al. 2006) or Hatha yoga (Benvenutti et al. 2017). Similarly, large-scale prospective studies that allow the temporal order between HPA axis disturbances and PTSD incidence to be established are warranted in order to confirm that such alterations are integral to the symptom development rather than a mere consequence of being chronically ill.

Second, there is no data on the long-term trajectory of cortisol after patients have undergone TF-CBT. Collecting such data could be important since a number of studies in depression and anxiety disorders have reported significant changes in cortisol at follow-up rather than at the end of treatment (Fischer and Zilcha-Mano under review; Laufer et al. 2018), indicating that therapeutic changes in biological systems may emerge with a time delay. Supporting this notion, a recent study in patients with anxiety disorders provided initial evidence that pre- to post-treatment changes in *FKBP5* methylation were strongly associated with treatment outcome at six-month follow-up but not at post-treatment (Roberts et al. 2019). Future studies aiming to illuminate the interaction of HPA axis functioning at the genetic, epigenetic and endocrine level with treatment response should monitor potential changes beyond psychotherapeutic treatment, i.e., by including follow-up assessments. Moreover, these follow-up assessments should be conducted at multiple time points to enhance the understanding of the time frame in which alterations occur.

Third, all (epi-)genetic studies have exclusively studied war-related PTSD, and in relation to this have almost exclusively examined men. As such, it is unclear whether the findings extend to women, who are not only more frequently affected by PTSD but are also more likely to be exposed to other types of trauma (e.g., sexual assault). Further studies are thus necessary to extend these important findings to women samples. In addition, it might be worthwhile to explore the impact of sex hormones on the development and maintenance of PTSD symptoms. Indeed, psychotherapy studies in the context of anxiety disorders have already demonstrated that sex hormone concentrations affect treatment outcomes (Graham et al. 2018). Given that TF-CBT is based on the same principles as gold standard treatments for anxiety disorders (i.e., fear extinction), it is conceivable that the same effects might be observable in PTSD.

Nevertheless, when taken together, it is feasible that a subset of individuals with PTSD, namely those with a specific genetic make-up and epigenetic alterations within HPA axis-relevant genes, present with a hypocortisolaemic pattern and more persistent PTSD symptoms that do not improve sufficiently after TF-CBT. Initial attempts at augmenting TF-CBT by administering glucocorticoids to patients with PTSD have already been undertaken (see e.g. de Quervain et al. 2017). Likewise, the development of drugs targeting epigenetic alterations in PTSD is conceivable and has begun to be implemented in animals (e.g., drugs targeting DNA methyltransferases, Zannas et al. 2015); however, this needs to be preceded by a more comprehensive understanding of the role of these processes in PTSD. Multilevel studies, that is, studies which integrate markers of the HPA axis at the genetic, epigenetic, and endocrine level, are essential in order to achieve a comprehensive characterisation of the proposed subgroup.
